# Clinical implementation of intensity modulated proton therapy for testicular seminoma

**DOI:** 10.2340/1651-226X.2025.43848

**Published:** 2025-09-25

**Authors:** Heidi S. Rønde, Jesper F. Kallehauge, Morten Høyer, Anne Birgitte Als, Mads Agerbæk, Jakob Lauritsen, Peter M. Petersen, Lars Dysager, Camilla J.S. Kronborg

**Affiliations:** a Danish Centre for Particle Therapy, Aarhus University Hospital, Aarhus, Denmark; b Department of Oncology, Aarhus University Hospital, Aarhus, Denmark; c Department of Oncology, Rigshospitalet, Copenhagen, Denmark; d Department of Oncology, Odense University Hospital, Odense, Denmark

**Keywords:** testicular seminoma, IMPT, stage IIa seminoma, proton therapy, secondary cancers, pencil beam scanning

## Abstract

**Background and purpose:**

We have previously shown that proton therapy results in considerable reduced doses to abdominal organs at risk (OAR) which likely reduces the patient’s risk of a second malignant tumor, which is vital for this young population with favourable prognosis. Here, we present dosimetric results after implementing intensity modulated proton therapy (IMPT) as a national standard for seminoma.

**Patient/material and methods:**

Thirty patients with stage IIA and IIB (< 3 cm) seminoma were treated with five-field robustly multi-field optimised (MFO) proton therapy to 20–24 Gy (relative biological effectiveness [RBE]) to the dog-leg retroperitoneal volume followed by a boost of 10–16 Gy (RBE) to the nodal metastasis. Control CTs were performed routinely, and target coverage evaluated. A standard two cone-beam CT (CBCT) set-up strategy with four match structures was developed, enabling implementation of a standard adaptive scheme.

**Results:**

The median clinical target volume (CTV-E) length in the craniocaudal direction was 26.9 cm, with a median volume of 551.4 cm3. Target coverage V_95%_ = 100% for the nominal plan and V_95%_ ≥ 98% for worst-case scenarios were fulfilled for all treatment plans and the 46 recalculated plans on control CTs. Kidney V_17Gy_ was 0–6% and mean kidney dose 0–6 Gy across all plans. Bowel bag V_15Gy_ was 194–698 cm3. All other OAR showed low doses.

Four patients had replans (1–2 per patient). The median time for our image guidance (IG) strategy was 14:07 min across all patients with two CBCTs.

**Interpretation:**

We have established a robust setup for treatment planning, IG strategy, treatment delivery and adequate response to replanning. Therefore, we suggest considering IMPT for testicular seminoma whenever available.

## Introduction

Patients with seminoma and indication for radiotherapy are in general younger men with a favourable prognosis. Attempt to reduce the integral dose, the risk of second malignancy and vascular morbidity is therefore of utmost importance.

Patients with limited stage retroperitoneal involvement have traditionally been treated with photons [1, 2] to a target covering retroperitoneal (para-aortic, paracaval, interaorto-caval, precaval and pre-aortic) and ipsilateral internal and external iliac lymph nodes regions also known as the ‘dog-leg’ [3].

In general, acute toxicities with photon treatment are mild [2, 3] while few patients experience late toxicities like erectile dysfunction [4], neurological [5], gastrointestinal and urogenital events as late as up to 30 years after treatment [6]. Importantly, some studies have shown a statistically significant increased relative risk (RR) of secondary cancer after radiotherapy of up to 2.0 (RR) for this patient group when treated with photon therapy [7, 8].

Previous dose planning studies have compared traditional photon therapy (3D conformal and IMRT) to proton treatment (passive/double scatter and uniform scanning). All have found a lower dose to surrounding organs at risk (OAR) with proton therapy, and both Efstathiou et al. and Simone et al. indicate that the model-based risk of secondary cancer is reduced with proton therapy [1, 9, 10].

Recent studies by Pasalic et al. from 2020, Maxwell et al. from 2023 and Pursley et al. from 2024 compared proton therapy (passive scatter and [pencil] beam scanning [PBS]) to photon therapy (3D conformal, Intensity Modulated Radiation Therapy (IMRT) and Volumetric Modulated Arc Therapy (VMAT)) and have confirmed the dosimetric advantage of proton therapy for seminoma patients [2, 11, 12]. Based on these observations and our own pre-implementation simulation study [13] we implemented proton therapy for these patients in September 2021 and have since then worked on establishing a strategy for planning and delivery of multi-field optimised intensity modulated proton therapy (MFO-IMPT) of seminoma stage IIA and IIB (< 3 cm).

The aim of this study was to verify that the dose to OAR could be kept low after clinical implementation, to describe a robust setup for treatment planning and image guidance (IG) strategy, and to suggest OAR optimisation criteria for MFO-IMPT of seminoma. Here we present the results of the first 30 patients treated with IMPT.

## Patients/material and methods

### Patients

**Figure 1:**
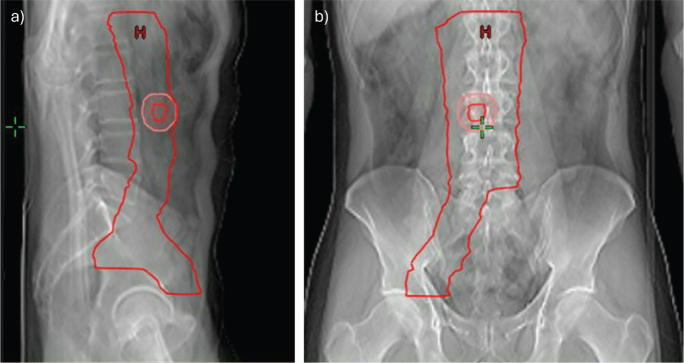
Extent of the target CTV-E (red) in (a) sagittal and (b) coronal section. Example of GTV-B (red) and CTV-B (pink) are also shown.

According to the national guidelines of the Danish Testicular Cancer Group (DaTeCa), patients with International Germ Cell Cancer Collaborative Group (IGCCCG) stage IIA seminoma (retroperitoneal or iliacal lymph node metastasis < 2 cm) or IIB (solitary lymph node up to 3 cm) should receive radiotherapy to the retroperitoneal and iliacal space [14]. All treated patients had recurrences after active surveillance of stage I seminoma corresponding to stage IIA or IIB (< 3 cm). Median age at the time of treatment was 45 years (range: 25–71). Twenty-one patients had 20 Gy (relative biological effectiveness, RBE) in 10 fractions and 9 had 24 Gy (RBE) in 12 fractions to the elective clinical target volume (CTV-E). According to the national guidelines [14], both regimes are acceptable and chosen per the clinician’s discretion. Further, for sequential boost to the pathological lymph node with a margin of 10 mm (CTV-B), 24 patients received 10 Gy (RBE) in five fractions, four were planned for 16 Gy (RBE) in eight fractions and two had no boost plan since the pathological lymph node was resected as a diagnostic procedure. Seventeen patients had left sided and 13 right sided metastasis. (See Table 1 for patient characteristics.) Figure 1 displays the extension of the target.

**Table 1:** Patient characteristics.

Characteristics	Numbers
Age (median, range)	45 (25–71) years
CTV-E volume (median, range)	551 (365–805) ccm
Dose (elective area)	
- 20Gy/10fx	21
- 24Gy/12fx	9
Dose (boost – pathological lymph node)	
- ^10Gy/5fx^	24
- ^16Gy/8fx^	4
- ^No boost^	2
Laterality	
- ^Left-sided^	17
- ^Right-sided^	13
Control CT per patient, n	
- ^Total^	46
- ^Median^	1
- Range	1–3

In brief, for delineation of the target the following margins were used: 8 mm to vena cava laterally on the right side, 12 mm anterior for aorta and vena cava, 17 mm laterally from aorta. Shaped to muscle and bones. Minor, visible lymph nodes should be included. For iliac vessels a 7 mm margin was added and 15 mm anterior-laterally for the external iliac vein. The craniocaudal boundaries were 15 mm cranial of the renal artery to the superior part of the acetabulum.

### Proton therapy dose planning

For simulation, a CT scan in a supine position with a knee-feet fixation and the patient’s arms on the chest was acquired. All scans were conducted with 2 mm slice thickness without contrast, followed by a CT scan with intravenous contrast. There was no use bladder filling protocols.

Dose planning followed the description in Rønde et al. from 2023 [13] on the planning CT without contrast (Supplementary Figure S1 show an example of the field arrangement). Plans were robustly MFO with 14 scenarios (5 mm setup error; 3.5% range uncertainty) in Eclipse (Varian Medical Systems v13.7 and v16). A constant RBE factor of 1.1 was used. Patients were treated on a Varian ProBeam machine with PBS. The plans consisted of five fields – three posterior covering the entire target length and two anterior covering only the most caudal pelvic part of the target. For the posterior fields a range shifter of 3 or 5 cm was used. OAR were delineated according to RTOG guidelines [15, 16], including body contour, bladder, duodenum, pancreas, stomach, bowel bag, spinal cord and kidneys.

### Dose to OAR

We aimed for as low as possible doses for the following dose volume histogram (DVH) objective metrics: bladder V_15Gy_ (%), bowel bag V_15Gy_ (cm3), duodenum V_15Gy_ (cm3), pancreas V_15Gy_ (cm3) and stomach V_15Gy_ (cm3), kidneys (right and left respectively) V_17Gy_ (%) and the mean dose, spinal cord D_0.03 cm3_ (Gy) and mean dose to body contour.

To guide the optimisation process, we collected OAR doses from the pre-implementation study and optimised after 10, 20 and 30 patients. Boost plans were excluded due to the heterogeneity between patients of localisation and extension of the pathological lymph node.

### Treatment delivery and IG strategy

The daily IG strategy included two cone beam CTs (CBCTs) due to the targets being longer than the 20 cm field-of-view for the ProBeam imaging system. CBCTs were registered to the planning CT based on bony anatomy. First, a pelvic CBCT 3–5 cm caudal to the isocentre, secondly, an abdominal CBCT 3–5 cm cranial to the isocentre was acquired. For the caudal CBCT, a maximum of 1.5° for pitch, roll and rotation was allowed before a 3D match was conducted. Deviations of body surface and bony structures of up to 5 mm from corresponding structures on treatment plan CT were accepted. However, for vertebral column cranially, only 3 mm was allowed, to minimize the risk of cranial misalignment on the second CBCT. On the cranial CBCT, body surface and vertebral column should be within 5 mm; no registration or couch move was done. The patients were repositioned if IG requirements were not met. A final 2DkV image at the isocentre was acquired to check that the table was back in the isocentre position before treatment.

### Weekly CTs during the course of treatment

A control CT scan in the treatment position (2 mm slice thickness, no contrast) was performed routinely during the course of treatment. Initially once weekly for the first 15 patients, subsequently once during the beginning of the course of treatment. The control CT was co-registered (6D rigid) with the planning CT, and the treatment plan was recalculated on the control CT, including ± 3.5% calibration curve error. Target coverage was evaluated for V_95%_ > 98% for dose to the CTV-E to determine if replanning was necessary. The daily CBCTs were also checked to ensure the comparability between the control CT and the daily positioning.

### Adaptive therapy – ART

Based on this strategy, an adaptive scheme was implemented after treating the first 15 patients. This included a systematic evaluation of match of body surface and bones, number of repositionings and changes in bowel gas. Three consecutive remarks of the same deviation would lead to offline checks/calculations to evaluate the need for a control CT and replanning. The systematic evaluation was performed daily by the radiation therapists during the IG strategy and the offline check by medical physicists.

## Results

Thirty patients were referred and treated between September 2021 and December 2024 and thus included in the study. The median length of the elective CTV-E was 26.9 cm (range: 21.8–32.0) in the craniocaudal direction with a median volume of 551.4 cm3 (365.4–805.4). In total, 46 control CTs were made with a median of 1 (1–3) per patient.

### Target coverage

All treatment plans fulfilled the target coverage of V_95%_ = 100% for the nominal plan and V_95%_ ≥ 98% for the worst-case scenario. Likewise, the worst-case scenario (V_95%_ ≥ 98%) was fulfilled for all dose plans recalculated on the 46 control CT scans.

### Dose to OAR

For the kidneys left and right, respectively, we found V_17Gy_ to be 0–6%, and the kidney mean dose was within 0–6 Gy across both 20 Gy and 24 Gy plans. Bowel bag V_15Gy_ was within 194–698 cm3. Duodenal V_15Gy_ was 1–85 cm3, pancreas V_15Gy_ 0–71 cm3, stomach V_15Gy_ 0–8 cm3 and bladder V_15Gy_ 0–15%. Near max (D_0.03 cm3_) for spinal cord was 10–20 Gy. A full list of doses to all OAR is summarised in Table 2.

**Table 2:** Results – median (range).

OAR	Dose metric	IMPT–20Gy	IMPT–24 Gy
Body contour	Mean [Gy]	1.5 (1.2–2.1)	1.8 (1.5–3.0)
Bladder	V15Gy [%]	1.2 (0–7.8)	1.5 (0–15.1)
Duodenum	V15Gy [cm3]	35.9 (1.1–84.8)	44.4 (25.5–80.5)
Pancreas	V15Gy [cm3]	32.3 (0–63.1)	34.9 (0.0–70.5)
Stomach	V15Gy [cm3]	0.0 (0.0–3.7)	0.0 (0.0–8.1)
Bowel bag	v15Gy [cm3]	428.9 (194.1–614.5)	462.2 (310.1–697.7)
Spinal cord	near max [0.03 cm3]	12.9 (9.8–18.2)	13.2 (11.8–19.9)
Kidney L	V17Gy [%]	0.0 (0.0–6.1)	0.4 (0.0–2.6)
Kidney L	Mean [Gy]	1.8 (0.5–4.1)	2.4 (0.0–2.6)
Kidney R	V17Gy [%]	0.6 (0.0–3.0)	2.2 (0.0–5.0)
Kidney R	Mean [Gy]	2.2 (0.8–5.6)	2.6 (1.3–5.7)

OAR: organs at risk; IMPT: intensity modulated proton therapy.

For future references, these OAR dose intervals could be used as optimisation criteria.

### IG strategy and treatment time

**Figure 2:**
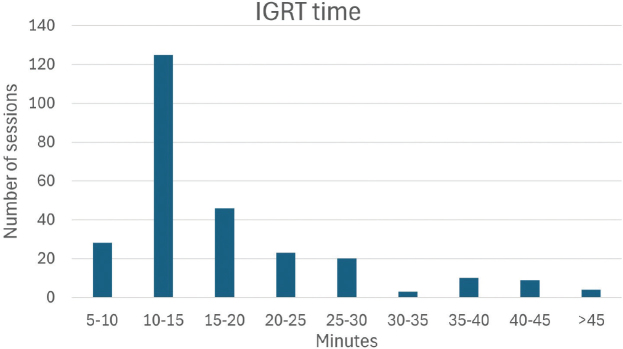
Distribution of IGRT times (in minutes) across all treatment fractions across all patients with two CBCTs.

The median time for our IG strategy including repositioning was 14:07 min (06:50–48:00) across all patients with two CBCTs (from beginning of first CBCT to onset of first spot). Figure 2 displays the distribution between fractions. The five fields were delivered in a median of 13.2 min (interquartile range [IQR]: 10.3–22.4), including insertion of the range shifter. Following the implementation of the adaptive scheme, two patients had no repositionings, six had one repositioning once during the course of treatment and five had multiple repositionings one, three or four times during the course of treatment.

### Replanning

Five replans were done, with one patient having two replans and three patients having one. Replanning was not performed based on the control CTs which all met the criteria of V_95%_ ≥ 98%. Instead, they were requested based on the daily CBCTs which showed larger changes in bowel gas requiring a change in the anterior beam angles or patients difficult to position and therefore a need for a more robust dose plan taking daily variation in for example the anterior body outline into account.

## Discussion and conclusion

We have demonstrated a robust treatment planning and IG strategy for the first 30 patients with seminoma treated with IMPT. The target dose coverage and robust evaluation (14 scenarios) fulfilled our standard criteria of V_95%_ ≥ 98%, and the implemented IG and control strategy led to a low number of replans. The time required for the IG strategy was considered acceptable given the complexity of the target.

Further we have shown that doses to OAR are comparable with the OAR doses of our pre-implementation study [13] and suggest OAR dose optimisation criteria for future proton planning.

Treatment plan guidelines have evolved over time and target volumes today are generally smaller than what was previously accepted. Therefore, a direct comparison of the pre-implementation OAR doses to the current clinical OAR doses is not feasible.

The number of publications with proton therapy used for treatment of testicular seminoma is limited. Based on models, proton therapy for testicular seminoma could be beneficial due to its superior dosimetric profile entailing a lower risk of secondary cancer, which is crucial for this population of younger men with good prognosis and long expected remaining life. This has been supported by a couple of newer publications with proton treatment of 5–24 patients compared to photon plans [2, 11, 12]. (Supplementary Table S1 lists a summary of proton studies.)

For most cancer types, treated with radiotherapy, dose constraints exist to guide the balance of an acceptable risk of both acute and late toxicities. In absence or when constraints can easily be achieved the ALARA (as-low-as-reasonably-achievable) principle is used [17] to strive to keep the dose to surrounding tissue low, even when low doses are prescribed. To avoid too much variation as to what is ‘reasonable’ in the context of proton therapy for seminoma, we undertook an approach using doses obtained in the pre-implementation study and updated them prospectively after the first 10, 20 and now 30 patients to adapt and strive to keep the dose to surrounding OAR as low as possible acknowledging a continuous learning curve. With photon treatment V_17Gy_ and mean dose was a national standard constraint for kidneys and therefore also used for proton treatment [18]. Near max (D_0.03 cm3_) for spinal cord being a serial organ is important. For bowel bag, V_15Gy_ has been suggested [19, 20] for which reason we also chose this as a reasonable measure for bowel bag, as well as for the bladder, duodenum, pancreas and stomach. For bladder it is standard to look at a percentage volume whereas an absolute volume is standard for the other OAR.

The studies by Pasalic et al. [2], Maxwell et al. [11] and Pursley et al. [12] all used 1–2 posterior fields. We used three posterior and two anterior (only most caudally) to cover the long target as described in Rønde et al. [13]. With IMPT as used in this study, it is feasible to use multiple fields which improves conformality [21]. Further, this makes the plans robust against smaller anatomical changes to body surface, and bowel gas which is seen by the few number of replans.

This study lacks prospective registration of acute toxicity and the use of supportive care, such as antiemetics. This would have allowed for greater insight into whether decreasing OAR doses indeed leads to a more favourable toxicity profile [11].

In line with the recommendation of Maxwell and Pursley to use proton therapy for testicular seminoma [11, 12], we have implemented proton therapy as a national standard. We have shown that low OAR doses were maintained with the clinical implementation of IMPT and presented OAR optimization criteria to be used as a reference for future studies. Further, we have shown a robust setup for treatment planning, suggestions for OAR optimisation criteria, IG strategy, treatment delivery and reactions to replans when needed. On this basis, we suggest considering IMPT for testicular seminoma when proton therapy is available, while acknowledging that not everyone has access to it.

## Supplementary Material



## Data Availability

Patient specific data cannot be shared due to the Danish legislation. Technical data can be shared upon reasonable request.
