# Supplementary material for: Clinical implementation of intensity modulated proton therapy for testicular seminoma
Source: Acta Oncol. 2025 Sep 25;64:43848. doi: 10.2340/1651-226X.2025.43848 (PMC12490103; doi:10.2340/1651-226X.2025.43848)
Supplement: Supplementary file 1 [file AO-64-43848-s1.pdf]

Supplementary material has been published as submitted. It has not been copyedited, or typeset by Acta Oncologica

Supplementary Material

Figure S1:

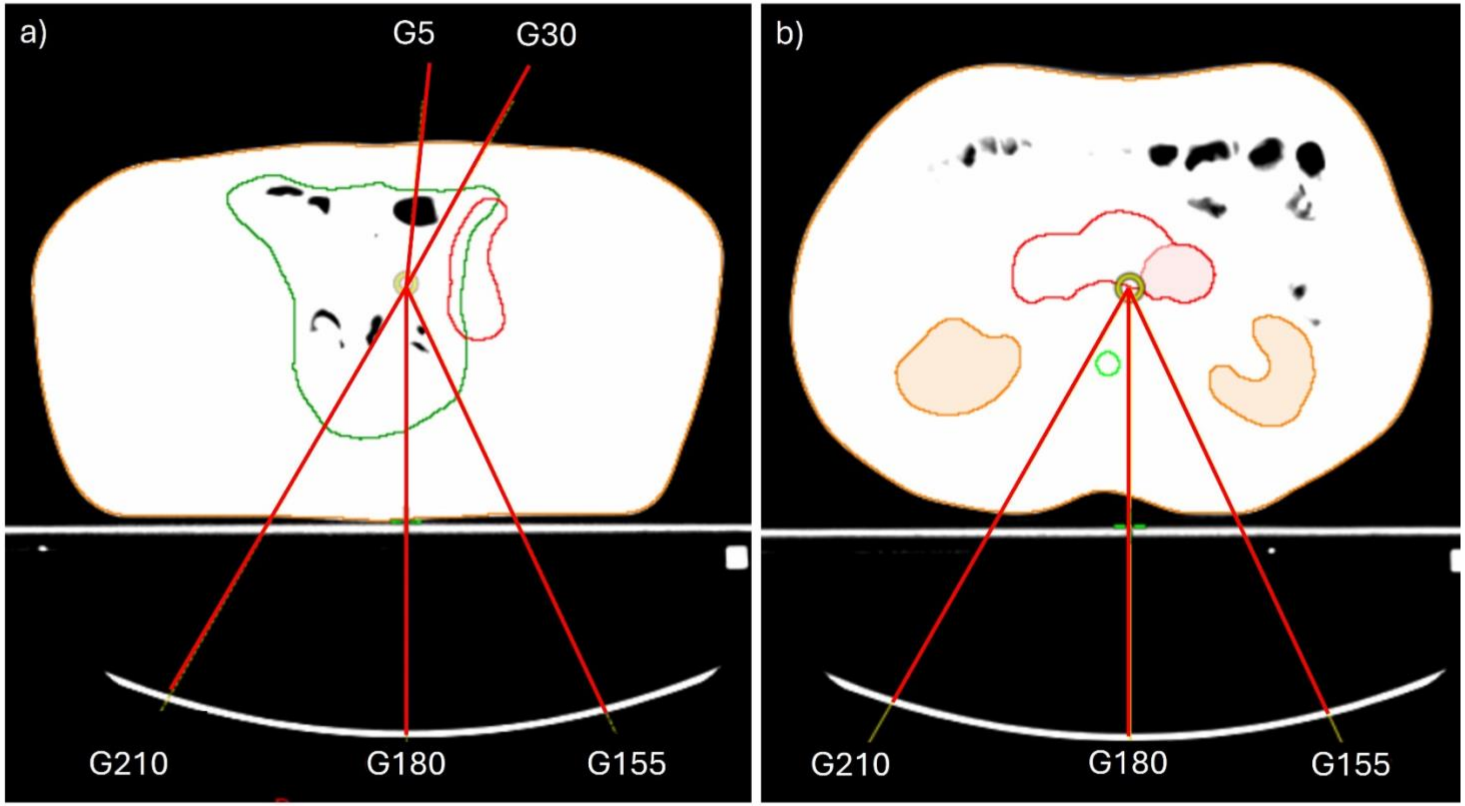

Example of beam arrangement: a) caudally and b) cranially.
CTV-E (red), CTV-B (pink), bowel bag (green), kidneys (orange), spinal cord (green).

Table S1: Studies on proton therapy for testicular seminoma

Author (year)	Patient number	Technique Photon/proton	Secondary cancer risk#	Toxicities*	Conclusion
Simone 2012 Ref no 1	10	Photon: 3D AP-PA fields Proton: Scanned PA field	Yes	n/a	Significant sparing of most critical OARs examined with protons. Reduced modelled secondary cancer risk with protons.
Efstathiou 2012 Ref no 10	10	Photon: 3D AP-PA fields Proton: Non scanning PA field & AP-PA fields	Yes	n/a	Significant sparing of most critical OARs examined with protons. Reduced modelled secondary cancer risk with protons.
Hoppe 2013 Ref no 9	2	Photon: 3D (AP-PA) & IMRT (5-7 fields) Proton: Double-scatter & Uniform-scanning (1-2 PA fields)	No	n/a	Reduced dose to normal tissue with protons. May translate to reduced toxicities and reduced risk of secondary cancers with protons.
Pasalic 2020 Ref no 2	55 (11 protons)	Photon: 3D AP-PA fields Proton: Passive scatter & scanning beam (1-2 PA fields)	Yes^	Yes	Excellent clinical outcomes with protons. Lower rates of acute diarrhea but higher rates of acute dermatitis. Reduced dose to OAR with protons. No in-field secondary malignancies with protons.
Rønde 2023 Ref no 13	10	Photon: IMRT (3-4 fields) & VMAT (2 arc) Proton: IMPT (5 fields)	Yes	n/a	Significant sparing of most critical OARs examined with protons. Reduced modelled secondary cancer risk with protons.
Maxwell 2023 Ref no 11	24	Photon: 3D AP-PA, IMRT & VMAT Proton: Passive scatter; Uniform scanning & PBS	Yes	Yes	Cancer control and toxicity outcomes with protons are consistent with existing photon-based literature. Protons may be associated with a significant lover risk of secondary cancers. Recommend proton therapy for testicular seminoma.
Pursley 2024 Ref no 12	10 (5 protons)	Photons: 3D AP-PA & VMAT (1-2 arc) Protons: PBS (1 PA field)	Yes	n/a	Protons show potential to reduce toxicities. Reduced modelled secondary cancer risk with protons. Recommend proton therapy for testicular seminoma.
Rønde 2025	30	Protons: IMPT (5 fields)	No	n/a	Low dose to OAR with suggested optimisation criteria for future proton planning. Recommend proton therapy for testicular seminoma.

#Modelled risk of secondary cancers
*Registered toxicity data
^Prospective registry
AP-PA: Anteriorposterior – Postrioranterior
PBS: Pencil beam scanning
OAR: Organs at risk
